# Treatment planning and evaluation of gated radiotherapy in left-sided breast cancer patients using the Catalyst^TM^/Sentinel^TM^ system for deep inspiration breath-hold (DIBH)

**DOI:** 10.1186/s13014-016-0716-5

**Published:** 2016-10-26

**Authors:** S. Schönecker, F. Walter, P. Freislederer, C. Marisch, H. Scheithauer, N. Harbeck, S. Corradini, C. Belka

**Affiliations:** 1 Department of Radiation Oncology, LMU University, Marchioninistraße 15, 81377 Munich, Germany; 2 Medical Clinic and Policlinic I, LMU University, Munich, Germany; 3 Breast Center, Department of Obstetrics and Gynecology, LMU University, Munich, Germany

**Keywords:** Surface scanner, Left-sided, Breast cancer, Cardiac toxicity, Deep inspiration breath-hold (DIBH), Audio-visual guided, Catalyst^TM^

## Abstract

**Background:**

There is a potential for adverse cardiovascular effects in long-term breast cancer survivors following adjuvant radiotherapy (RT). For this purpose, the deep inspiration breath-hold technique (DIBH) has been introduced into clinical practice, to maximally reduce the radiation dose to the heart. However, there are a variety of DIBH delivery techniques, patient positioning and visual patient feedback mechanisms. The aim of the present study was to evaluate the application of radiotherapy in DIBH using the Catalyst^TM^/Sentinel^TM^ system, with a special emphasis on treatment planning and dosimetric plan comparison in free breathing (FB) and DIBH.

**Patients and methods:**

A total of 13 patients with left-sided breast cancer following breast conserving surgery were included in this prospective clinical trial. For treatment application the Catalyst^TM^/Sentinel^TM^ system (C-RAD AB, Uppsala, Sweden) was used and gating control was performed by an audio-visual patient feedback system. CT and surface data were acquired in FB and DIBH and dual treatment plans were created using Pencil Beam and Collapsed Cone Convolution. Dosimetric output parameters of organs at risk were compared using Wilcoxon signed-rank test. Central lung distance (CLD) was retrieved from iView^TM^ portal images during treatment delivery.

**Results:**

The system contains a laser surface scanner (Sentinel^TM^) and an optical surface scanner (Catalyst^TM^) interconnected to the LINAC systems via a gating interface and allows for a continuous and touchless surface scanning. Overall, 225 treatment fractions with audio-visual guidance were completed without any substantial difficulties. Following initial patient training and treatment setup, radiotherapy in DIBH with the Catalyst^TM^/Sentinel^TM^ system was time-efficient and reliable. Following dual treatment planning for all patients, nine of 13 patients were treated in DIBH. In these patients, the reduction of the mean heart dose for DIBH compared to FB was 52 % (2.73 to 1.31 Gy; *p* = 0.011). The maximum doses to the heart and LAD were reduced by 59 % (47.90 to 19.74 Gy; *p* = 0.008) and 75 % (38.55 to 9.66 Gy; *p* = 0.008), respectively. In six of the nine patients the heart completely moved out of the treatment field by DIBH. The standard deviation of the CLD varied between 0.12 and 0.29 cm (mean: 0.16 cm).

**Conclusion:**

The Catalyst^TM^/Sentinel^TM^ system enabled a fast and reliable application and surveillance of DIBH in daily clinical routine. Furthermore, the present data show that using the DIBH technique during RT could significantly reduce high dose areas and mean doses to the heart.

**Trial registration:**

DRKS: DRKS00010929 registered on 5. August 2016.

## Introduction

Whole breast radiotherapy after breast-conserving surgery is a fundamental cornerstone in the treatment of early breast cancer and has been shown to halve the risk of local recurrence and reduce the annual breast cancer death rate by about one sixth [1]. Furthermore, modern systemic treatment regimens containing substances such as anthracyclines or the antibody trastuzumab, have significantly improved progression free survival and overall survival in patients with early breast cancer [2, 3]. However, these substances have been reported to induce relevant cardiotoxicity and pose a risk for development of life-threatening congestive heart failure [4]. The combination of anthracyclines and/or trastuzumab with RT may synergistically influence the risk for cardiac toxicity [5].

Recent data suggest, that even lower radiation doses to the heart may play a relevant role for the development of late cardiac toxicity after breast cancer treatment [6–8]. Radiation exposure of the heart can result in coronary artery disease, congestive heart failure, valvular heart disease, pericardial disease, conduction abnormalities and sudden cardiac death [9]. However, neither the exact pathogenesis, nor the responsible anatomical substructures of the heart influencing late cardiac toxicity, or the precise dose–response relationship are well understood. At present, different clinical approaches were developed in order to decrease the radiation dose to the heart in breast cancer treatment: Technologies like forward-planned and inverse-planned IMRT (including Tangential IMRT) and helical tomotherapy [10–13] have been shown to reduce the high dose exposure of the heart but often at the expense of significantly increasing low dose exposures.

Another approach is the application of respiratory-gated and breath-hold RT, such as deep inspiration breath-hold (DIBH) [14–19], which probably allows for a real dose reduction without any increases in low dose areas. Currently, several vendor specific systems are in use: for example the Active Breathing Control (ABC) device designed at William Beaumont Hospital, Michigan [20]; the Varian Real-time Position Management system (RPM^TM^) (Varian Medical Systems, Palo Alto, CA, USA [21]), or the AlignRT system of Vision RT Ltd, London, UK, [22]. Another system is the Respiratory Gating Laser System AZ-733 V of the manufacturer Anzai using a contactless laser sensor (Anzai Medical Co., Ltd, Tokyo, Japan), or the breast belt system [23]. In the present study the Catalyst^TM^/Sentinel^TM^ system of C-RAD AB, Uppsala, Sweden was used. The system consists of a laser-based surface scanner (Sentinel^TM^) and an optical-based surface scanner (Catalyst^TM^) interconnected to the LINAC systems via the Response^TM^ gating interface. The system allows for a continuous surface scanning, LINAC triggering and includes an active patient feedback system (audio-visual guidance). In contrast to other vendor specific systems (ABC, RPM), there is no device or “box” that has to be positioned on the patient.

The objective of the present study was to evaluate treatment planning and dosimetric plan comparison in free breathing (FB) and DIBH, and the application of radiotherapy in DIBH using the Catalyst^TM^/Sentinel^TM^ system in left-sided breast cancer patients in clinical daily routine.

## Materials and methods

**Fig. 1:**
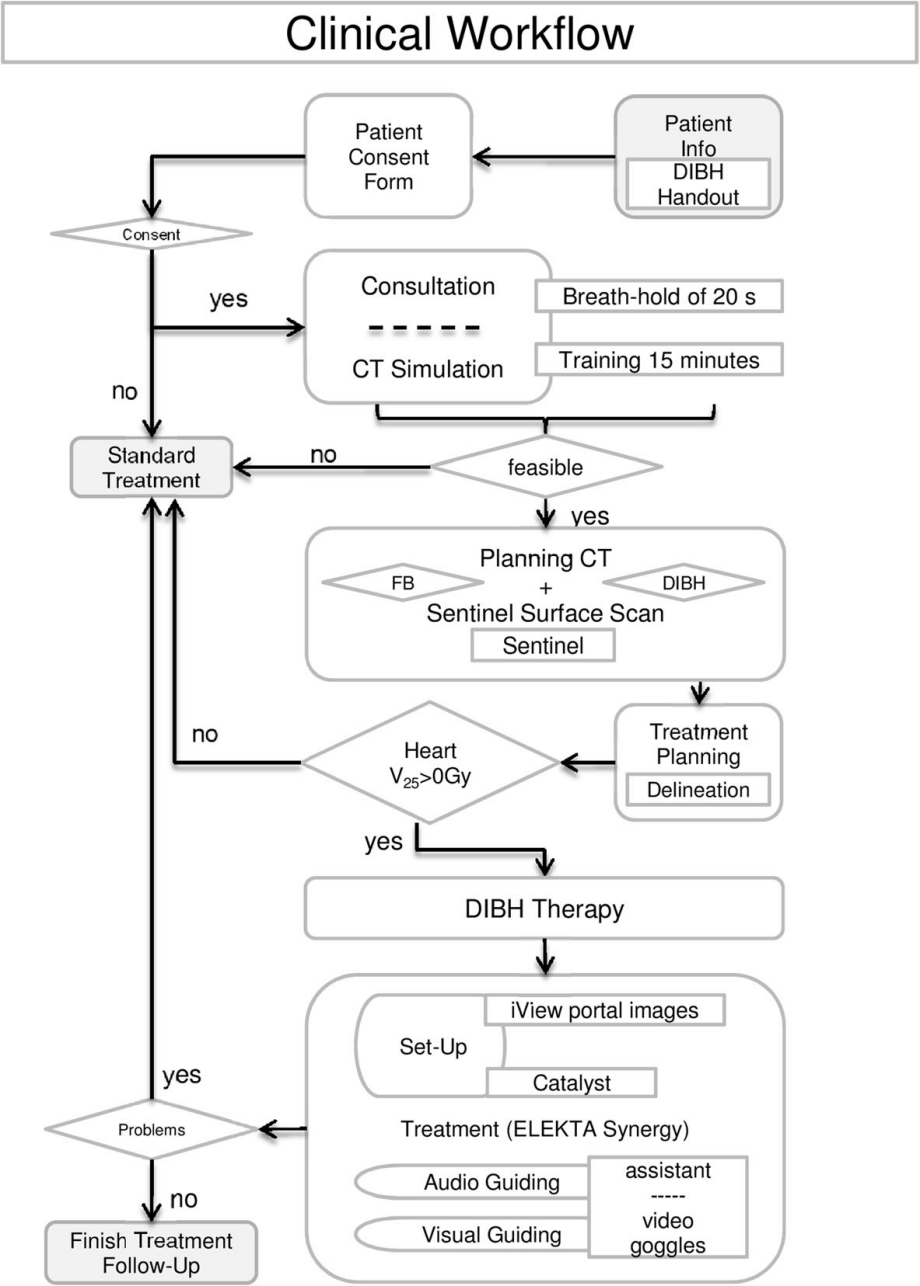
Schematic overview of the clinical workflow

The prospective study was performed in accordance with the Declaration of Helsinki and was approved by the ethical committee of the LMU medical faculty (22.10.2013, No. 496–12). Inclusion criteria were informed consent, left-sided breast cancer, breast conserving surgery and patient compliance (ability of breath-hold for 20 s). A total of 13 left-sided breast cancer patients, with a mean age of 46.9 years (range: 36–63 years) were included in the study. The workflow is depicted in Fig. 1, showing our clinical routine with the initial training phase one and two, the acquisition of the two planning CTs using the Sentinel^TM^ scanner, the dual treatment planning, the clinical decision making and the setup/treatment with the audio-visual gating and the Catalyst^TM^ system in the treatment room.

### Treatment planning

All patients received two planning CT scans, one in free breathing (FB) and one in DIBH, each with a slice thickness of 3 mm. The heart and the left anterior descending artery (LAD) were delineated according to the CT-based atlas by Feng et al. [24]. In order to reduce the inter-observer variability, the same physician delineated all organs at risk (OAR). Target volume delineation, treatment planning and dose concepts were made according to the German interdisciplinary consensus- based guideline (“S3 guideline on “Diagnosis, Therapy, and Follow-up of Breast Cancer” [25]) and the practical guidelines of the Breast Cancer Expert Panel of the German Society of Radiation Oncology (DEGRO) [1] and applied for both, FB and DIBH. The clinical target volume (CTV) included all remaining ipsilateral breast tissue, including the deep fascia but not the underlying muscle or overlaying skin. The margin for the planning target volume (PTV) was 0.5-1.0 cm covering the entire breast and taking into account adjustments on the medial and lateral borders. The prescribed dose was 50.0 Gy (2.0 Gy/d).

Treatment planning was performed using the Oncentra 4.3 (Nucletron, Veenendaal, Netherlands) software. To ensure consistency, all treatment plans were made by the same physicist. The algorithm used for dose calculation was Collapsed Cone Convolution (CCC) with a 3 mm × 3 mm calculation grid. For dosimetric plan comparison all plans were additionally recalculated in Pencil Beam Convolution (PBC).

The following parameters were derived from the DVHs of the different treatment plans: the mean and the maximum dose to the heart (D_mean,_ D_max_), and the relative volume of the heart receiving at least 5, 10, 15, 20, 25, 35 and 45 Gy (V_5_–V_45_). The dosimetric parameter V_25_ of the heart was used as a cut-off value in order to decide if a patient was treated using the DIBH manoeuvre or not. Patients presenting with low cardiac dose exposure (V_25_ of 0 % in FB) would most likely not benefit from RT in DIBH. The dose to 2 % of the LAD volume (D_2%_) was recorded as a robust estimator for the maximum dose to the LAD and the D_2%_ of the 10 mm expanded LAD. Further parameters included: The PTV coverage described by the volume receiving 95 % of the prescribed dose (V_95%_), the mean doses to right and left lung, as well as the volume of the left lung receiving more than 20 Gy (D_mean_, V_20_). Wilcoxon signed-rank tests were used to estimate statistical significance of differences between FB and DIBH.

### Radiation treatment using the Catalyst^TM^/Sentinel^TM^ system

**Fig. 2:**
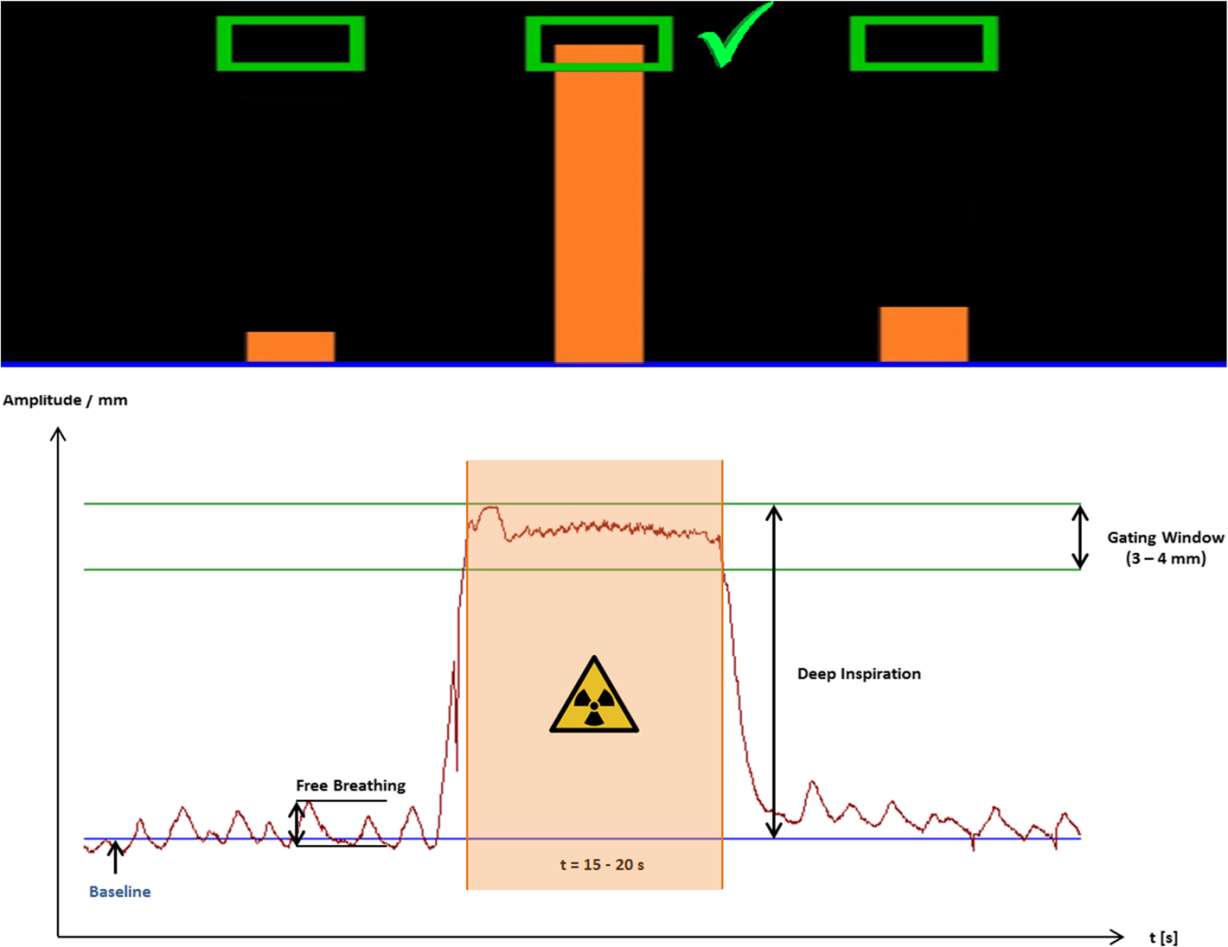
Visual feedback of the breathing position for the patient: gating window (*green box*) and breathing position (*orange bar*). Following an audio command the patient is requested to locate (breath) the orange bar into the predefined gating window. Original motion signal of a breathing curve depicting automated beam gating

The Sentinel^TM^ system is a laser-based (λ = 635–690 nm) optical surface scanning system, which is used during CT acquisition in order to create a reference surface scan in free breathing and to record the breathing patterns including the deep inspiration amplitude during the deep inspiration manoeuvre. The gating window was initially set arbitrarily to 4 mm at the level of stable deep-inspiration (see example of gating window in Fig. 2). The amplitude of the gating window should not exceed the amplitude of free breathing. When a stable and constant breath hold was observed during initial training, the gating window was stepwise reduced to a maximum of 2 mm during the training at the CT. If the gating window was adjusted too small during initial training, some patients had problems in keeping the inspiration level within the predefined range. For visual feedback of the breathing position for the patient, Cinemizer® OLED (Carl Zeiss, Oberkochen, Germany) video goggles were used. Throughout the DIBH CT acquisition for treatment planning, the patient was asked to keep a maximum comfortable deep inspiration. The patient was requested to locate (breath) the orange indicator bar into the predefined gating window (green box) following an audio command signal of the radiation therapist in order to start CT acquisition or radiotherapy treatment (see Fig. 2). CT acquisition time ranged between 10 and 15 s, depending on the length of the thorax of each patient. In general, the pitch of the CT couch has been set to 15 mm/s. The gaiting window was defined due to the deep inspiration amplitude during the deep inspiration manoeuvre, detected through the Sentinel system in the CT room. The measurement of the sentinel system was then transferred to the Catalyst system which is interconnected to the LINAC.

**Fig. 3:**
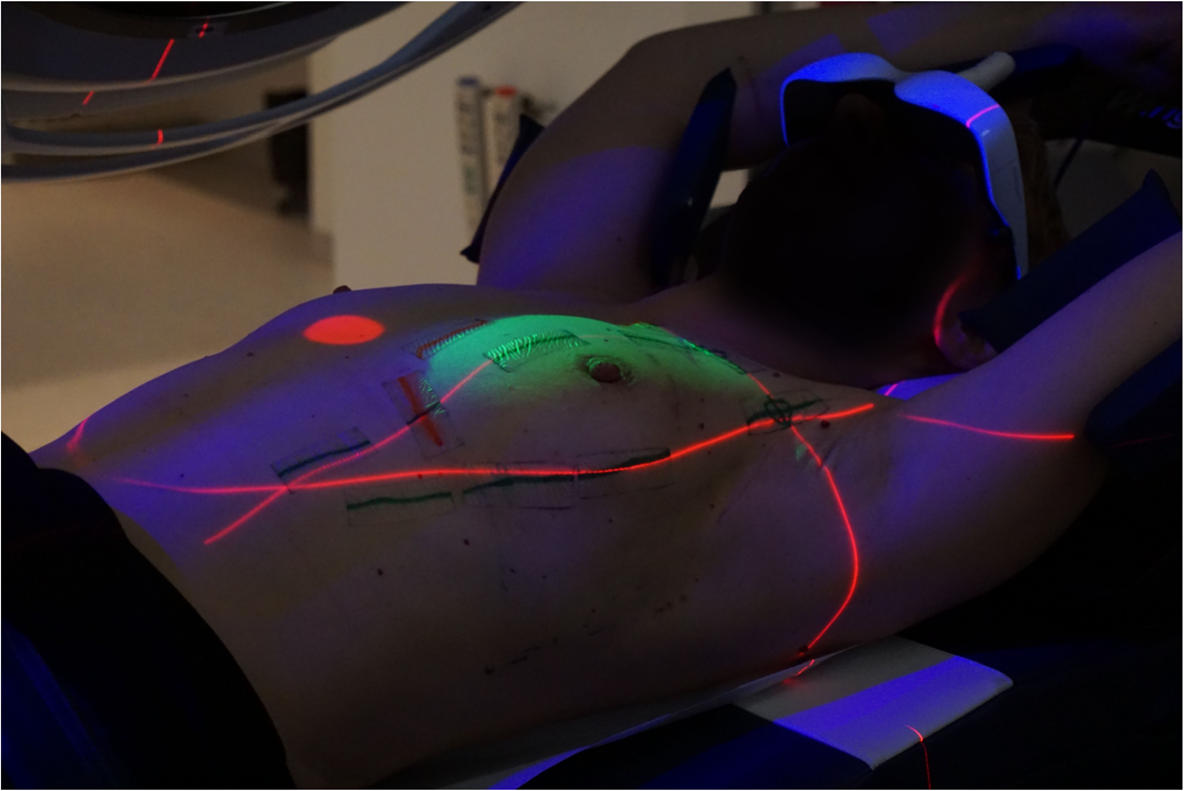
Typical patient treatment setup: the trigger point on the sternum of the patient (*red dot*), the treatment beam visualized by light (*green*), the room lasers (*red lines*), the scanning light of the catalyst (*blue*) and video goggles

The Catalyst^TM^ system works through an optical surface scanning with LED lights (blue: λ = 450 nm) and reprojection captured by a CCD camera (green: λ = 528 nm; red: λ = 624 nm), which provides target position control during set-up and treatment (Fig. 3). For 3D surface reconstruction, the system uses a non-rigid body algorithm to calculate the distance between the surface and the iso-center and using the principle of optical triangulation. The system works with a frame rate of 200 frames per second. For further details regarding the Catalyst^TM^ system, see: Freislederer et al. [26] or the manufacturer's website.

During treatment delivery, a spot on the patient’s sternum, monitored by the Catalyst^TM^ optical surface scanner was used to initiate treatment delivery automatically using the Elekta Response^TM^ interface. The size of the spot on the patient's surface is a circle with the radius of r = 20 mm. The system averages all measurement points in that radius in order to acquire a reproducible gating spot. The tracking of this spot is ensured by the system's software and has been tested in advance.

For the first treatment, patient positioning and stable deep breath-hold were verified using the Sentinel^TM^ surface scan acquired during CT simulation as a reference. A new reference scan was taken manually in the Catalyst^TM^ system after the patient has been repositioned during the first treatment fraction. The new reference image is necessary due to the fact that during the planning CT, the image was acquired using the Sentinel laser based-system while the patient was lying in the CT gantry in the reference position. The new surface scan during the first treatment fraction was acquired using the Catalyst system and has a far better resolution due to the fact that the Catalyst system works with another acquisition method, using optical visible light. Furthermore, the patient's surface will be better visible, as there is no interference with the CT-gantry, which leads to better positioning results because of an even better and clearer reference image. The new reference image was automatically set as the new reference image used for the following treatment fractions. Furthermore following our clinical routine, manually triggered iView^TM^ portal images (Elekta AB, Sweden) were acquired once a week during deep inspiration breath-hold in order to verify the patient positioning. In these images the distance from the deep field edge to the thoracic wall (central lung distance, CLD) was measured and documented.

## Results

Dual treatment planning was performed for all 13 patients. Four patients presented with a low cardiac dose (V_25_ of the heart: 0 %) (Table 1), and did therefore not receive radiotherapy in DIBH. The remaining nine patients had higher cardiac doses (Table 2) and were treated in DIBH RT.

**Table 1:** Parameters derived from DHVs (FB and DIBH) of four patients treated in FB. Prescribed dose was 50.0 Gy in 2.0 Gy fractions. Algorithm: CCC

	FB		DIBH		∆
Heart					
D_max_ (Gy)	10,94 ± 2,48	[7,81–13,31]	6,37 ± 1,63	[4,89–8,12]	*p* = 0,068
D_mean_ (Gy)	1,14 ± 0,15	[1,00–1,28]	0,97 ± 0,15	[0,82–1,18]	*p* = 0,068
V_5_ (%)	0,68 ± 0,36	[0,22–1,06]	0,23 ± 0,23	[0,01–0,47]	*p* = 0,068
V_10_ (%)	0,05 ± 0,04	[0,00–0,10]	0,00 ± 0,01	[0,00–0,01]	*p* = 0,109
V_15_ (%)	0,01 ± 0,01	[0,00–0,01]	0,00 ± 0,00	[0,00–0,00]	*p* = 0,157
V_25_ (%)	0,00 ± 0,00	[0,00–0,00]	0,00 ± 0,00	[0,00–0,00]	*p* = 1,000
LAD					
D_mean_ (Gy)	3,45 ± 1,16	[2,19–4,97]	2,46 ± 0,66	[1,82–3,07]	*p* = 0,068
D_2%_ (Gy)	6,11 ± 1,91	[3,82–8,47]	4,90 ± 1,69	[3,41–6,62]	*p* = 0,465
Ipsilateral lung					
D_mean_ (Gy)	6,38 ± 1,66	[5,10–8,76]	6,01 ± 1,76	[4,09–8,12]	*p* = 0,465
V_20_ (%)	10,81 ± 3,90	[8,14–16,44]	9,88 ± 3,87	[5,69–14,60]	*p* = 0,273

**Table 2:** Parameters derived from DHVs (FB and DIBH) of nine patients treated in DIBH. Prescribed dose was 50.0 Gy in 2.0 Gy fractions. Algorithm: CCC

	FB		DIBH		∆
Heart					
D_max_ (Gy)	47,90 ± 1,39	[45,38–50,18]	19,74 ± 15,52	[6,41–48,23]	*p* = 0,008
D_mean_ (Gy)	2,73 ± 1,40	[1,44–5,81]	1,31 ± 0,15	[1,08–1,49]	*p* = 0,011
V_5_ (%)	6,75 ± 4,39	[3,11–15,90]	1,18 ± 0,77	[0,17–2,55]	*p* = 0,008
V_10_ (%)	4,12 ± 3,45	[1,53–11,64]	0,26 ± 0,39	[0,00–1,12]	*p* = 0,008
V_15_ (%)	3,39 ± 3,18	[1,15–10,40]	0,14 ± 0,25	[0,00–0,74]	*p* = 0,008
V_20_ (%)	2,92 ± 2,95	[0,92–9,48]	0,09 ± 0,18	[0,00–0,53]	*p* = 0,008
V_25_ (%)	2,55 ± 2,74	[0,69–8,68]	0,06 ± 0,14	[0,00–0,42]	*p* = 0,008
V_35_ (%)	1,86 ± 2,30	[0,37–7,04]	0,03 ± 0,08	[0,00–0,23]	*p* = 0,008
V_45_ (%)	0,84 ± 1,40	[0,03–4,12]	0,01 ± 0,02	[0,00–0,07]	*p* = 0,008
LAD					
D_mean_ (Gy)	18,91 ± 9,78	[4,82–33,26]	4,19 ± 1,52	[2,53–6,83]	*p* = 0,008
D_2%_ (Gy)	38,55 ± 12,40	[9,02–48,71]	9,66 ± 6,30	[3,64–22,12]	*p* = 0,008
D_2%_ 10 mm expanded (Gy)	48,63 ± 1,54	[44,97–50,60]	29,98 ± 15,52	[6,85–47,41]	*p* = 0,008
Ipsilateral lung					
D_mean_ (Gy)	8,01 ± 2,02	[5,50–11,43]	6,45 ± 1,31	[5,06–7,98]	*p* = 0,008
V_20_ (%)	14,87 ± 4,41	[9,06–22,03]	10,96 ± 3,10	[6,87–47,41]	*p* = 0,008
Contralateral lung					
D_mean_ (Gy)	0,48 ± 0,17	[0,32–0,76]	0,51 ± 0,13	[0,32–0,67]	*p* = 0,953
PTV					
V_95_ (%)	81,78 ± 2,92	[77,32–85,95]	81,04 ± 4,95	[68,81–85,13]	*p* = 0,953

### Training and treatment delivery using the Catalyst^TM^/Sentinel^TM^ system

If the patient was eligible for the study (ability of breath-hold for 20 s) a brief training was performed during patient consultation in order to get familiar with the DIBH manoeuvre. Thereafter, all patients received a short introduction into technical details and the hardware (video goggles), followed by a profound 15-min hands-on training immediately prior to CT acquisition. Following this training, all 13 patients were able to complete CT simulation without facing any substantial problems. The average gating window over all patients was 3.1 mm. The mean deep inspiration breathing amplitude measured at the xiphoid with the Sentinel^TM^ system was 16.5 mm (range 11.9 – 24.0 mm). The nine patients successfully completed overall 225 radiotherapy fractions with good compliance and no interruption. Overall, the workflow operated time-efficiently. Despite the additional time reserved for patient training, dual CT acquisition and treatment planning, no significant extra treatment time has been observed during treatment delivery. The most time-consuming part accounting for one or two extra minutes was the set-up of the visual feedback, for which the patient had to position the video goggles prior to treatment delivery. The maximum time of one radiation field was about 15 s (~150 Monitor Units (MUs), Dose Rate ~ 660 MUs/min). During the gantry rotation of the LINAC in between two treatment fields, all patients were able to have enough rest to maintain a stable breath hold during the subsequent treatment field. In conclusion, no substantial additional overall treatment time has been observed.

### Verification of deep inspiration breath-hold

To verify precise dose delivery in DIBH a total of 55 iView^TM^ portal images have been acquired over the course of treatment. There were no major deviations requiring additional imaging. The standard deviations of the CLD were varying between 0.12 and 0.29 cm. The mean value of the standard deviations over all patients was 0.16 cm, which is an acceptable value for clinical routine.

### Doses exposure of the heart and LAD

Mean PTV volumes were 1065 ccm for FB and 1059 ccm for DIBH (*p* = 0.859). Regarding patients treated in DIBH (Table 2), the mean heart dose was 2.73 Gy (range: 1.44–5.81 Gy) in FB as compared to 1.31 Gy (range: 1.09–1.49 Gy) in DIBH. Overall, the mean heart dose could be decreased by 52 % in patients treated in DIBH (*p* = 0.011). The mean dose to the LAD artery was 18.91 Gy (4.82–33.26 Gy) in FB versus 4.19 Gy (2.53–6.83 Gy) in DIBH, respectively. That corresponds to a significant mean dose reduction of 78 % (*p* = 0.008). V_25_ of the whole heart was 2.55 % (0.69 – 8.68 %) in FB as compared to 0.06 % (0.00–0.14 %) in DIBH. In six patients the V_25_ could be reduced to 0 % using DIBH.

Concerning maximal dose exposure to the heart in FB, the maximum heart dose was 47.90 Gy (45.38–50.18 Gy) and the dose to 2 % of the volume of the LAD, was 38.55 Gy (9.02–48.71 Gy). Through the use of the DIBH manoeuvre the maximum dose to the heart could be reduced to 19.74 Gy (6.41–48.23 Gy) and the D_2%_ of the LAD was reduced to 9.66 Gy (3.64–22.12 Gy) in DIBH. This corresponds to a highly significant dose reduction of 59 % (*p* = 0.008) for the maximum heart dose and 75 % (*p* = 0.008) for the D_2%_ of the LAD.

### Dose exposure of the lung

Since DIBH strongly affects the anatomy of the surrounding lung, potential changes of the dose exposure to the ipsi- and contralateral lung were analysed. The mean dose to the left lung could be decreased in all patients, while the dose to the right lung was not significantly changed (*p* = 0.953) with a D_mean_ of the right lung in DIBH of 0.51 Gy (0.32–0.67 Gy). Concerning the left lung, a mean dose of 8.01 Gy (5.50–11.43 Gy) was detected in FB and 6.45 Gy (5.06–7.98 Gy) in DIBH, which corresponds to a significant absolute reduction of 1.56 Gy (*p* = 0.008). Regarding the dose distribution of the left lung volume receiving more than 20 Gy (V_20_), the V_20_ could be decreased by 26 % (14.87 to 10.96 %, *p* = 0.008).

### Collapsed cone vs. pencil beam

**Fig. 4:**
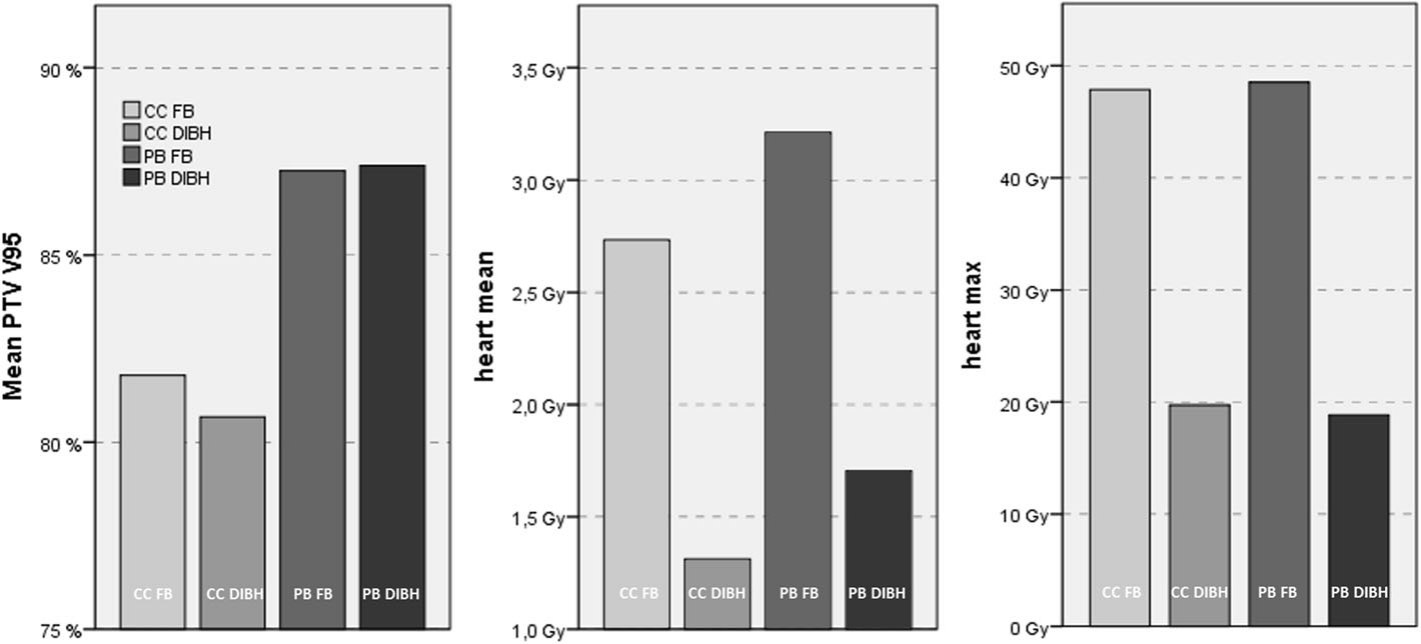
Mean PTV V95%, heart mean and maximum dose comparison between pencil beam and collapsed cone for FB and DIBH of nine patients treated in DIBH

As commonly known in medical physics, dose calculations are heavily influenced by the used algorithms. Therefore, we performed dose calculations using two different algorithms (CCC and PBC) and compared dose distributions. As shown in Fig. 4 there are certain dosimetric differences concerning PBC. Mean V_95%_ of the PTV for FB was 81.78 / 87.25 % (CCC / PBC) versus 80.82 / 87.46 % for DIBH. V_20_ of ipsilateral lung was 14.87 / 14.25 % for FB and 10.96 / 10.13 % for DIBH. Maximum heart dose in FB was 47.90 / 48.53 Gy as compared to 19.74 / 18.87 Gy in DIBH. All differences were significant (heart max (DIBH): *p* = 0.038; others: *p* = 0.008).

## Discussion

DIBH is suggested to be a robust method to reduce the radiation dose to the heart. The present data show that using the DIBH technique during RT could significantly reduce high dose areas and mean doses to the heart. This may be of particular clinical value for long-term breast cancer survivors in order to reduce the risk of late cardiac morbidity and mortality [27].

In the present study, DIBH resulted in a reduction of the mean heart radiation dose of absolute 52 % (2.73 to 1.31 Gy). Similarly, Hjelstuen et al. [18], Vikström et al. [28] and Stranzl et al. [17] found mean cardiac dose reductions between 50 and 56 % through the application of DIBH as compared to conventional RT. The latest study by Darby et al. [29] showed that rates of major coronary events increased linearly with the mean dose to the heart by 7.4 % per Gy. Using these risk models [29], the rate of major coronary events could theoretically be decreased by roughly 10 % through the use of DIBH.

Although the effect of DIBH in reducing the mean heart dose is consistent with other studies, the present planning study shows even better simulated results regarding decreased dose exposure to the LAD. In the present study, dose reduction of the LAD by application of the DIBH manoeuvre showed significantly decreased mean and D_2%_ doses to the LAD of absolute 78 and 75 %, respectably. In contrast, the studies of Hjelstuen [18] and Vikström [28] documented a mean LAD dose reduction of 56–65 % and a maximum dose reduction by 39 (D_2%_) to 57 % (D_max_). Obviously, there are certain limitations of this way of comparing and evaluating radiation doses of organs at risk from different studies. Besides the different definitions of “maximum dose” (D_2%_ versus D_max_), there might be a heterogeneity caused by the different organ at risk delineations of the heart. While some studies delineated the entire heart [30] as organ at risk, others limited the contours to the left ventricle [31, 32], the pericardium [32], or the LAD alone [33, 34]. To minimize the bias in the present planning study, the heart and LAD were delineated in accordance with the CT-based heart atlas developed by Feng et al. [24]. Furthermore, the algorithm used for dose calculation can significantly influence final dose distribution and the magnitude of dose reduction to the heart. To compare the different dose distributions in the present planning study, all plans were calculated using both algorithms, PBC and CCC. As one would expect, the mean V_95%_ of the PTV and the mean and maximum heart doses showed divergent simulated results depending on the applied algorithm. As pointed out by Koeck et al. [35], PBC is known to underestimate low dose areas, whereas high dose areas are overestimated [36]. This fact might further be influenced by the individual delineation of the target volume. The delineation of the PTV up to the skin surface may account for variations and inaccuracies in the expected external beam surface dose calculations and influence PTV coverage. Taken together, the comparison of dose distributions from different studies should be interpreted with caution.

The present study delivered DIBH RT using the Catalyst^TM^/Sentinel^TM^ system (C-RAD AB, Uppsala, Sweden). While the Catalyst^TM^ system provided the target position control during set-up and treatment through the optical surface scanning, the Sentinel^TM^ system recorded the deep inspiration amplitude through a laser based optical surface scanning system. An active patient feedback system (audio-visual guidance) was used to reach the optimal breathing position. The individually chosen gating window of 2–4 mm proved to be practicable in clinical routine and is within the range of previous studies [17, 18, 37]. If the gating window was adjusted too small during initial training, some patients had problems in keeping the inspiration level within the predefined range. With longer training, an individual reduction of the gating window seems feasible and should be evaluated in further studies.

The present study assessed the practicality and feasibility for radiotherapy in DIBH by means of Catalyst^TM^/Sentinel^TM^ system in clinical practice. The workflow operates time-efficiently and reliably, leading to good patient compliance. In clinical routine, most patients were treated in 15–20 min time slots, depending on the number of radiation fields. Verification of the DIBH with iView^TM^ portal images has shown excellent results with a reproducible breath hold level. The mean value of the standard deviations of the CLD over all patients was 0.16 cm. However, for a more conclusive and elaborated verification of external surface position reproducibility (e.g. 3D localizations of patient rib cage compared with the surface information) additional investigations are needed.

Compared to the Active Breathing Control (ABC) device, the Catalyst^TM^/Sentinel^TM^ system is more comfortable and less invasive, even though the reproducibility is limited due to irradiation within a 2-4 mm range (gating window) and the theoretical possibility of breathing during irradiation. Other limitations of an optical system are problems in detecting the surface of the object, e.g. in regions with increased body hair, which is obviously negligible in treatment of female breast cancer patients. Furthermore, in obese patients the vision of the Sentinel^TM^ system in the CT room could be limited due to a prominent belly.

Unfortunately, we could not define a valid criterion to select patients who benefit from DIBH in terms of mean heart dose, before initiation of RT. Despite individual anatomic characteristics, in our patients a suitable parameter seems to be the V_25_ of the heart resulting from treatment planning in FB. In the present study, the V_25_ of the heart has been used as a cut-off value in order to decide if a patient was treated using the DIBH manoeuvre or not. A V_25_ of 0 % was an indicator for low cardiac dose exposure and for whether the heart was within the treatment fields or not.

The present study was very useful in providing an insight into an alternative gating modality using the Catalyst^TM^/Sentinel^TM^ surface scanning system for DIBH. Concerning the clinical outcome and long-term benefit, it seems unlikely to make a general prevision of the magnitude of benefit regarding late cardiac toxicity for patients treated in DIBH. This correlation might be strongly influenced by a variety of factors, other than the solely effect of radiotherapy. On the one hand, additional systemic therapies (e.g. anthracycline, trastuzumab) may synergistically influence the incidence of cardiovascular disease (CVD), and on the other hand, CVD taken by itself is strongly influenced by cardiovascular risk factors. Conventional and influenceable risk factors are hypertension, hypercholesterolemia, increased body mass index, diabetes mellitus and smoking [38]. Therefore, additional primary and secondary prevention and risk reduction therapy for patients undergoing radiotherapy and presenting with risk factors for coronary and other atherosclerotic vascular disease could be beneficial for overall outcome [27]. In order to predict the individual treatment benefit from DIBH, further studies are needed with longer follow-up and better patient stratification. In this context, the use of screening strategies could help identifying patients that are more likely to benefit from DIBH RT [39]. Due to the above-mentioned limitations and in absence of long-term follow-up, to date, we cannot extrapolate possible clinical benefits from the present study. However, taking into account the results of Darby et al. [29], we can hypothesize a significant clinical benefit for patients treated with DIBH due to the significant reduction of radiation exposure of the heart.

## Conclusion

The Catalyst^TM^/Sentinel^TM^ system (C-RAD) enabled a fast and reliable application and surveillance of DIBH in daily clinical routine. Furthermore, the present data show that using the DIBH technique during RT could significantly reduce high dose areas and mean doses to the heart in patients with left-sided breast cancer.

## Abbreviations

BCS: Breast-conserving therapy
CCC: Collapsed cone convolution
CLD: Central lung distance
CVD: Cardio vascular disease
DIBH: Deep inspiration breath-hold
DVH: Dose-volume histogram
FB: Free breathing
LAD: Left anterior descending artery
MU: Monitor unit
OAR: Organs at risk
PBC: Pencil beam convolution
PTV: Planning target volume
RT: Radiotherapy
